# Efficacy and safety of lenvatinib plus durvalumab combined with hepatic arterial infusion chemotherapy for unresectable intrahepatic cholangiocarcinoma

**DOI:** 10.3389/fimmu.2024.1397827

**Published:** 2024-05-10

**Authors:** Rongce Zhao, Jing Zhou, Zhaoxia Miao, Xinhao Xiong, Wei Wei, Shaohua Li, Rongping Guo

**Affiliations:** ^1^ Department of Liver Surgery, State Key Laboratory of Oncology in South China, Collaborative Innovation Center for Cancer Medicine, Sun Yat-sen University Cancer Center, Guangzhou, China; ^2^ Department of Pathology, State Key Laboratory of Oncology in South China, Collaborative Innovation Center for Cancer Medicine, Sun Yat-sen University Cancer Center, Guangzhou, China

**Keywords:** unresectable intrahepatic cholangiocarcinoma, hepatic arterial infusion chemotherapy, lenvatinib, durvalumab, FOLFOX

## Abstract

**Background:**

The prognosis for unresectable intrahepatic cholangiocarcinoma (ICC) is poor and the efficacy of traditional chemotherapy remains unsatisfactory. Hepatic arterial infusion chemotherapy (HAIC) with oxaliplatin, leucovorin, and 5-fluorouracil (FOLFOX) is effective in patients with unresectable ICC. In this study, we determined the preliminary clinical efficacy and safety of lenvatinib plus durvalumab combined with FOLFOX-HAIC in patients with untreated, unresectable ICC.

**Materials and methods:**

Between July 2021 and July 2023, patients with unresectable ICC who initially received lenvatinib plus durvalumab combined with FOLFOX-HAIC at the Sun Yat-Sen University Cancer Center (SYSUCC) were reviewed for eligibility. Efficacy was evaluated by tumor response rate and survival, and safety was assessed by the frequency of key adverse events (AEs).

**Results:**

A total of 28 eligible patients were enrolled. The objective response rates (ORRs) based on mRECIST and RECIST 1.1 criteria were 65.2% and 39.1%, respectively. The median OS was 17.9 months (95% CI, 5.7–30.1) and the median PFS was 11.9 months (95% CI, 6.7–17.1). Most patients (92.9%) experienced adverse events (AEs), whereas 46.5% (13/28) experienced grade 3 or 4 AEs.

**Conclusion:**

Lenvatinib plus durvalumab combined with FOLFOX-HAIC showed promising antitumor activity and manageable AEs in patients with treatment-naive unresectable ICC. This regimen may be suitable as a novel first-line treatment option for this patient population.

## Introduction

Cholangiocarcinoma is a heterogeneous spectrum of highly aggressive adenocarcinomas that arise from the biliary epithelium. It is categorized as intrahepatic cholangiocarcinoma (ICC) and extrahepatic cholangiocarcinoma (ECC) (1). ICC is the second most common primary liver cancer, accounting for up to 20% of all liver malignancies. Over the past 40 years, the incidence of ICC has increased more than 140% (2). Surgical resection is the only curative treatment; however, most patients are diagnosed with advanced disease when clinical symptoms appear hence lose the opportunity for surgery (3). The prognosis of the patients with unresectable ICC is very poor, with a median survival of 2.5–7.5 months in the absence of treatment (4).

Gemcitabine plus cisplatin (GC) is the first-line systemic chemotherapy recommended for unresectable biliary tract cancer; however, its survival benefits are limited as it has a median overall survival (OS) of only 11.7 months (5). Compared with systemic chemotherapy, hepatic arterial infusion chemotherapy (HAIC) enables the delivery of high concentrations of chemotherapeutic drugs directly into the liver with fewer systemic side effects (6, 7). Previous studies have indicated that HAIC has higher tumor control rates compared with systemic chemotherapy for advanced ICC (8, 9). The FOLFOX regimen has shown considerable efficacy and is recommended as a palliative treatment option for advanced ICC (10, 11). In a previous study, we demonstrated that FOLFOX-HAIC was safe and effective for treating unresectable ICC (12), which was consistent with the results of Cai et al. (13). This indicates the promising therapeutic effect of FOLFOX-HAIC for advanced ICC. Based on this evidence, FOLFOX-HAIC may be an effective treatment for advanced ICC.

Lenvatinib is a novel oral multikinase inhibitor that exerts antiangiogenic and direct antitumor effects (14). Based on the results of the REFLECT study, lenvatinib has been recommended as the first-line standard treatment for advanced hepatocellular carcinoma (HCC) by guidelines such as NCCN, ESMO, and CSCO (15). Recently, several studies have reported that lenvatinib exhibits remarkable efficacy in advanced bile duct cancer (BTC) (16–18).

Recently, the TOPAZ-1 trial showed that durvalumab, a programmed cell death 1 ligand 1 (PD-L1) antibody, combined with chemotherapy provides a significant survival benefit in the patients with locally advanced or metastatic BTC. Therefore, it is one of the preferred first-line regimens for advanced BTC (19).

In recent years, combination therapy with lenvatinib, PD-1/PD-L1 antibody, and FOLFOX-HAIC demonstrated significant efficacy in advanced HCC, which not only showed an improved objective response rate (ORR), but also significantly prolonged patient survival (20). However, the efficacy of this treatment in unresectable ICC has rarely been reported. In this study, we examined the efficacy and safety of lenvatinib, durvalumab, and FOLFOX-HAIC as first-line treatment for unresectable ICC.

## Materials and methods

This study was conducted according to the ethical guidelines of the Declaration of Helsinki and approved by the Ethics Committee (B2020-318-01) of Sun Yat-Sen University Cancer Center (SYSUCC; Guangzhou, China).

### Patients

Between July 2021 and July 2023, patients diagnosed with ICC who received lenvatinib, durvalumab, and FOLFOX-HAIC treatment in the Department of Liver Surgery of SYSUCC were reviewed for eligibility. The eligibility criteria were as follows: (1) ICC confirmed by clinical or histopathological evidence; (2) assessed by at least two experienced hepatobiliary surgeons as unsuitable for surgical resection; (3) 18 years or older and less than 75 years old; (4) adequate hepatic and renal function; (5) Eastern Cooperative Oncology Group Performance Status (ECOG PS) score of 0–1; and (6) at least one measurable target lesion that could be evaluated by Response Evaluation Criteria in Solid Tumors, version 1.1 (RECIST 1.1) and modified RECIST (mRECIST). Patients were excluded based on the following criteria: (1) previously received antitumor treatment; (2) history of other malignant tumors; (3) incomplete medical information; and (4) loss to follow-up.

### Treatment procedure

HAIC was performed every three weeks as described previously (21). Briefly, a microcatheter was placed in the main feeding hepatic artery and the following regimen was infused: 135 mg/m^2^ oxaliplatin from hour 0 to 3 on day 1; 400 mg/m^2^ leucovorin from hour 3 to 4.5 on day 1; 400 mg/m^2^ 5-fluorouracil bolus from hour 4.5 to 6.5 on day 1; and 2400mg/m^2^ 5-fluorouracil over 46 h from day 1 to day 3. Lenvatinib (The UK, Eisai Europe Co. Ltd.) was administered orally every day with a dose of 8 mg. Durvalumab was administered intravenously with a dose of 1000 mg every three weeks. The first administration of lenvatinib and durvalumab was within 7 days of HAIC initiation. Lenvatinib and durvalumab treatment continued after HAIC was discontinued until tumor progression or intolerable adverse reactions occurred.

### Data collection

All clinical baseline characteristics of the eligible patients were collected from medical records filed at SYSUCC, including gender, age, Child–Pugh score, the status of hepatitis virus infection, tumor size, tumor number, tumor distribution, vascular invasion, lymph node (LN) metastasis, distant metastasis, tumor–node–metastasis (TNM) stage, carbohydrate antigen 19-9 (CA19-9), white blood cell count, neutrophil (NE) count, hemoglobin (HGB), platelet (PLT) count, alanine transaminase (ALT), serum albumin levels (ALB), total bilirubin, prothrombin time, and creatinine (CRE).

### Follow-up and survival analyses

Each follow-up visit included a medical history taking, physical examination, laboratory tests, and contrast-enhanced computed tomography (CT) and/or magnetic resonance imaging (MRI) examination. The initial follow-up appointment was administered 6–8 weeks (2 cycles of HAIC) after the first treatment. This study was followed until November 1, 2023.

OS was defined as the interval from the date of the initial administration of HAIC to death from any cause. PFS was defined as the interval from the initial administration of HAIC to tumor progression or death from any cause, whichever occurred first. ORR was defined as the proportion of patients achieving either a complete response (CR) or a partial response (PR), which was maintained for at least four weeks from the first radiological confirmation. The disease control rate (DCR) was defined as the ORR plus the percentage of patients with stable disease (SD). Tumor response was assessed using Response Evaluation Criteria in Solid Tumors, version 1.1 (RECIST 1.1) and modified RECIST (mRECIST) (22, 23). Adverse events (AEs) were evaluated based on the National Cancer Institute Common Terminology Criteria for Adverse Events version (NCI-CTCAE) 4.03.

### Statistical analysis

Normally distributed variables were described as the mean ± standard error (SE) and nonnormal distributions were described as the median and interquartile range (IQR). Categorical variables were compared using the Pearson χ2 test or Fisher’s exact test. Continuous parametric variables were compared by the unpaired Student’s t-test, whereas for continuous nonparametric variables, the Mann–Whitney U test was used. The Kaplan–Meier method was used for survival analysis and the log-rank test was used for survival curve differences. Forward LR-based univariate and multivariate Cox regression analyses were used to identify independent predictive variables. A two-tailed p < 0.05 was considered statistically significant. All data analyses were performed using SPSS software, version 24.0 (SPSS Inc., Chicago, IL, USA).

## Results

### Patient characteristics

Between July 2021 and July 2023, 28 patients who met the inclusion criteria and agreed to be treated with lenvatinib, durvalumab, and FOLFOX-HAIC were included in this study. The clinical characteristics of these patients are shown in **Table 1**.

**Table 1:** Baseline clinical characteristics of the included patients (n = 28).

Characteristics	Patients
Gender	
Male	14 (50.0%)
Female	14 (50.0%)
Age	
Median (IQR)	60 (50.5–65)
Mean (SD)	56.2 (12.0)
Child)Pugh score	
5	25 (89.3%)
6	3 (10.7%)
Hepatitis virus infection	
HBV	10 (35.7%)
HCV	0
None	18 (64.3%)
Tumor size, cm	
Median (IQR)	8.1 (4.8–10.3)
Mean (SD)	7.7 (3.1)
≤5	7 (25.0%)
>5	21 (75.0%)
Tumor number	
Single	12 (42.9%)
Multiple	16 (57.1%)
Tumor distribution	
Uni-lobe	14 (50.0%)
Bi-lobe	14 (50.0%)
Vascular Invasion	
Absence	18 (64.3%)
Presence	10 (35.7%)
LN metastasis	
Absence	10 (35.7%)
Presence	18 (64.3%)
Distant metastasis	
Absence	16 (57.1%)
Presence	12 (42.9%)
TNM stage	
I	2 (7.1%)
II	6 (21.5%)
IIIb	8 (28.6%)
IV	12 (42.8%)
CA19-9, U/mL	
≤40	11 (39.3%)
>40	17 (60.7%)
WBC, median (IQR), ×10^9^/L	8.4 (6.8–9.9)
NE, median (IQR), ×10^9^/L	3.9 (5.4–7.4)
Hgb, median (IQR), g/L	119.5 (130.5–141.5)
PLT, median (IQR), ×10^9^/L	189.5 (271–300.5)
ALT, median (IQR), U/L	16.3 (28.6–47.3)
ALB, median (IQR), g/L	38.9 (45.3–46.9)
TBil, median (IQR), μmol/L	9.2 (12.4–16.8)
PT, median (IQR), seconds	10.6 (11.5–11.8)
CRE, median (IQR), μmol/L	59.1 (64.5–72.7)

IQR, interquartile range; SD, standard deviation; HBV, hepatitis B virus; HCV, hepatitis C virus; LN, lymph node; CA19-9, carbohydrate antigen 19-9; WBC, white blood cell; NE, neutrophil count; Hgb, hemoglobin; PLT, platelet count; ALT, alanine transaminase; ALB, albumin; TBil, total bilirubin; PT, prothrombin time; CRE, creatinine.

The population consisted of 14 males and 14 females, ranging in age from 30 to 73 years. Among the patients, all had Child– Pugh class A liver function, 10 (35.7%) had hepatitis B infection, the maximum tumor diameter was 10.3 cm, 16 (57.1%) had multiple lesions, 10 (35.7%) had macrovascular invasion, 18 (64.3%) had hilar LN metastasis, and 12 (42.9%) had distant metastasis.

### Tumor response and patient survival

**Figure 1:**
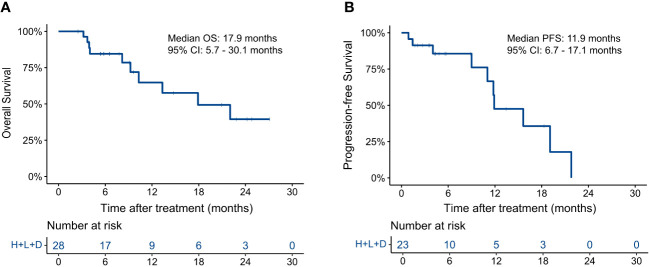
Kaplan–Meier curves for **(A)** overall survival and **(B)** progression-free survival.

A total of 74 HAIC cycles were performed during the study, with a median of 2.5 cycles (range, 1 to 4 cycles). At the end of follow-up, 10 patients had died (10/28, 35.7%), and 10 had disease progression (10/23, 43.5%). As shown in **Figure 1**, the median OS was 17.9 months (95% CI, 5.7–30.1) and the median PFS was 11.9 months (95% CI, 6.7–17.1). The 3-, 6-, and 12-month OS rates were 100.0%, 84.5%, and 64.8%, respectively. The 3-, 6-, and 12-m PFS rates were 91.3%, 85.6%, and 47.6%, respectively.

**Figure 2:**
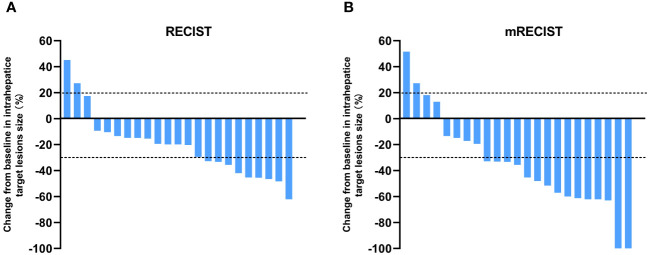
Best percentage changes from baseline in the size of the intrahepatic target lesions of patients receiving lenvatinib plus durvalumab combined with FOLFOX-HAIC. **(A)** RECIST1.1 was used to evaluate the image measurements before and after treatment. **(B)** Evaluated using mRECIST in patients with image measurements before and after treatment.

**Figure 3:**
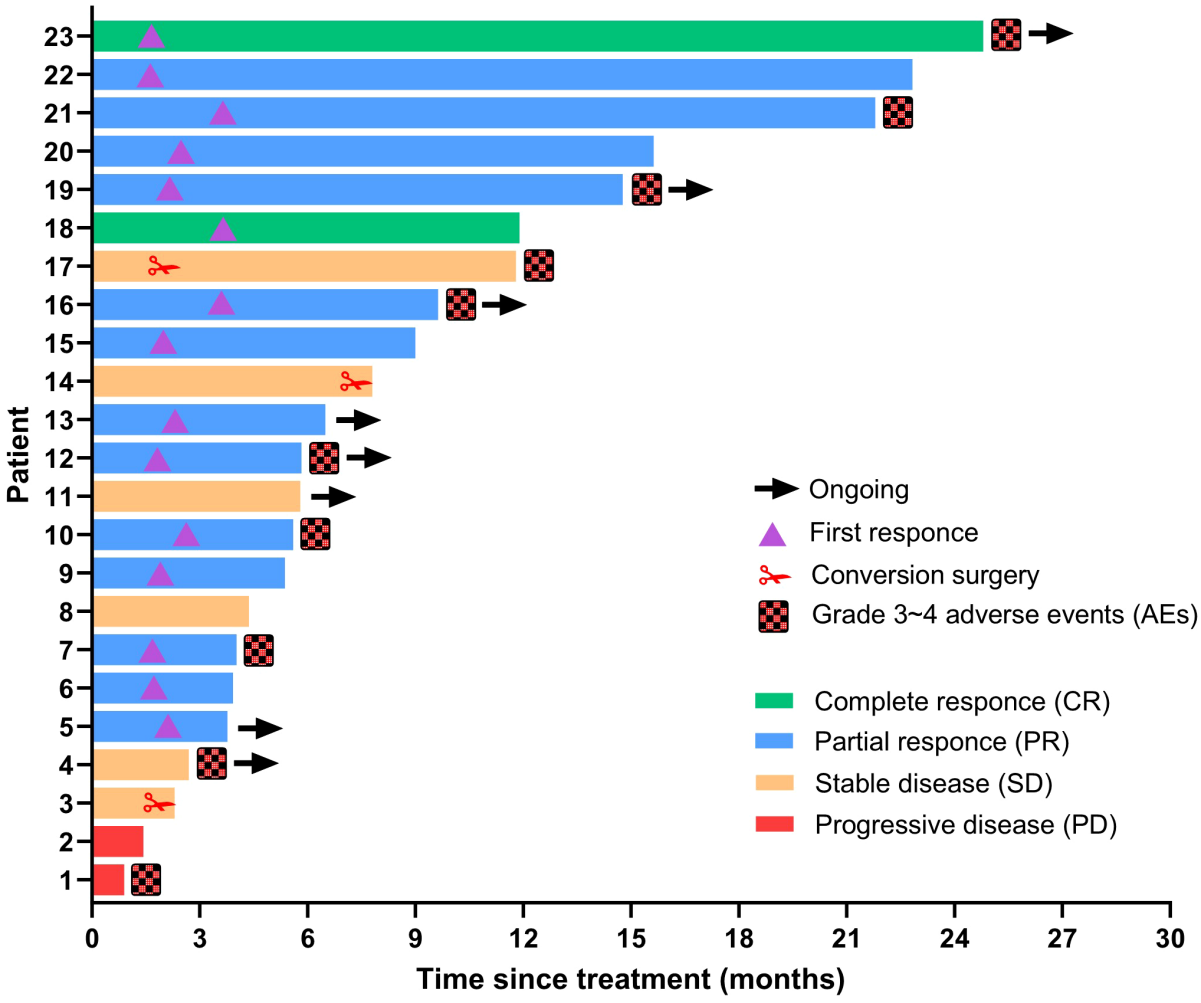
Duration of treatment and response assessments using mRECIST. The length of each bar represents the duration of treatment for each patient.

The tumor response outcomes are shown in **Table 2**. Of the 28 enrolled patients, 23 were evaluable for tumor response assessment. The ORRs based on the RECIST 1.1 and mRECIST criteria were 39.1% and 65.2%, respectively. Two patients (2/23, 8.7%) achieved a CR based on the mRECIST criteria and the DCR was 91.3%. The change in the intrahepatic target lesion size of the patients is shown in **Figure 2**. As shown in **Figure 3**, three patients exhibited tumor shrinkage or downstaging after treatment and eventually underwent radical liver tumor resection. Eight patients remained on treatment at the time of the follow-up cut-off. The median time to response (TTR) was 2.1 months (range, 1.6–4.2 months). The median duration of treatment was 5.8 months (range, 0.9–24.8 months).

**Table 2:** Tumor response (n = 23).

Characteristics	RECIST	mRECIST
Complete response (CR)	0	2 (8.7%)
Partial response (PR)	9 (39.1%)	13 (56.5%)
Stable disease (SD)	12 (52.2%)	6 (26.1%)
Progressive disease (PD)	2 (8.7%)	2 (8.7%)
Objective response rate (ORR)	39.1%	65.2%
Disease control rate (DCR)	91.3%	91.3%

RECIST, Response Evaluation Criteria in Solid Tumors; mRECIST, modified RECIST.

The prognostic factors of all clinical variables were analyzed by a univariate analysis, which showed that tumor response based on mRECIST (p = 0.038) and cycles of HAIC (p = 0.037) were significant risk factors for patient OS. CA19-9 (p = 0.075), vascular invasion (p = 0.077), and ALB (p = 0.065) appeared to exhibit a trend toward a difference; however, the p-value was not statistically significant, possibly because of the small sample size. Univariate analysis for PFS showed that tumor response based on RECIST (p = 0.043) and mRECIST (p = 0.039), as well as cycles of HAIC (p = 0.016), were significant risk factors. A multivariate Cox proportional analysis revealed that the cycles of HAIC (p = 0.016) were significant and an independent prognostic factor of PFS (**Supplementary Table 1**).

The OS of responders based on RECIST 1.1 appeared to be better than that of the non-responders, but there was no statistical difference (p = 0.053, HR = 0.151, 95% CI 0.017-1.314). The PFS of responders based on RECIST 1.1 was significantly better than that of nonresponder (p = 0.017, HR = 0.111, 95% CI 0.013–0.934). Moreover, the OS (p = 0.018, HR = 0.163, 95% CI 0.029–0.901) and PFS (p = 0.019, HR = 0.039, 95% CI 0.029–0.909) of responders based on the mRECIST criteria were better than those of the non-responders (**Supplementary Figure 1**).

### Adverse events and safety

There was one treatment-related death in this study. A 71-year-old female was diagnosed with bronchiectasis, type 2 diabetes, a history of cerebral infarction, and cholecystolithiasis. The patient developed a severe pulmonary infection after one cycle of FOLFOX-HAIC, lenvatinib plus durvalumab treatment, which was considered the main cause of death.

At the time of the first treatment cycle, three patients had dose modification of the HAIC agent because of hepatic dysfunction. No patients had dose modification because of AEs during subsequent treatment cycles. As shown in **Table 3**, the frequency of key AEs of all grades was 92.9% (26 AEs in 28 patients). Treatment-related AEs (TRAEs) are shown in **Table 3**. The most common grade 1–2 AEs included hypoalbuminemia (89.3%), anemia (67.9%), elevated ALT (64.3%), elevated CRE (64.3%), and pain (50.0%). The most common grade 3–4 AEs were elevated ALT (5.8%), anemia (7.1%), and pain (7.1%). Any grade liver dysfunction in most patients, such as hypoalbuminemia, elevated ALT, and hyperbilirubinemia, was primarily mild to moderate and returned to normal following treatment. Furthermore, specific abdominal pain associated with the HAIC of oxaliplatin occurred in 14 (50.0%) patients, but most pain was mild and tolerable. Severe pain was rare and can be relieved quickly by suspending the oxaliplatin infusion or the use of spasmolytics, after which drug administration continued.

**Table 3:** Summary of adverse events by severity.

Adverse event	Any grade	Grade 1-2	Grade 3	Grade 4	Treatment-related grade ≥ 3
Total	26 (92.9%)	26 (92.9%)	12 (42.9%)	1 (3.6%)	12 (42.9%)
Hypoalbuminemia	25 (89.3%)	25 (89.3%)	0	0	0
Anemia	19 (67.9%)	17 (60.7%)	2 (7.1%)	0	2 (7.1%)
ALT level elevated	18 (64.3%)	14 (50.0%)	4 (14.3%)	0	4 (14.3%)
CRE level elevated	18 (64.3%)	18 (64.3%)	0	0	0
Abdominal pain	14 (50.0%)	12 (42.9%)	2 (7.1%)	0	2 (7.1%)
Hyperbilirubinemia	12 (42.9%)	12 (42.9%)	0	0	0
Neutropenia	10 (35.7%)	9 (32.1%)	1 (3.6%)	0	1 (3.6%)
Thrombocytopenia	11 (39.3%)	8 (28.6%)	3 (10.7%)	0	3 (10.7%)
Leukocytopenia	9 (32.1%)	8 (28.6%)	1 (3.6%)	0	1 (3.6%)
PT prolonged	6 (21.4%)	6 (21.4%)	0	0	0
Bloating	6 (21.4%)	6 (21.4%)	0	0	0
Vomiting	6 (21.4%)	6 (21.4%)	0	0	0
Diarrhea	5 (17.9%)	5 (17.9%)	0	0	0
Insomnia	3 (10.7%)	3 (10.7%)	0	0	0
Rash	3 (10.7%)	3 (10.7%)	0	0	0
Dysuria	3 (10.7%)	3 (10.7%)	0	0	0
Poor appetite	3 (10.7%)	3 (10.7%)	0	0	0
Constipation	2 (7.1%)	2 (7.1%)	0	0	0
Cough	2 (7.1%)	2 (7.1%)	0	0	0
Pneumonia infection	2 (7.1%)	1 (3.6%)	0	1 (3.6%)	1 (3.6%)
Hiccup	1 (3.6%)	1 (3.6%)	0	0	0
Fever	1 (3.6%)	1 (3.6%)	0	0	0
Hypothyroidism	1 (3.6%)	1 (3.6%)	0	0	0
Hypertension	1 (3.6%)	1 (3.6%)	0	0	0
Cholecystitis	1 (3.6%)	1 (3.6%)	0	0	0
Cholangiolithiasis	1 (3.6%)	0	1 (3.6%)	0	1 (3.6%)
Arrhythmia	1 (3.6%)	1 (3.6%)	0	0	0
HA dissection	1 (3.6%)	1 (3.6%)	0	0	0
Phlebitis	1 (3.6%)	1 (3.6%)	0	0	0
Subphrenic abscess	1 (3.6%)	0	0	1 (3.6%)	1 (3.6%)

AE, adverse events; HAIC, hepatic arterial infusion chemotherapy; ALT, alanine transaminase; CRE, creatinine; PT, prothrombin time; HA, hepatic artery.

## Discussion

Our team has been focusing on the potential of FOLFOX-HAIC for the treatment of primary liver cancer, including HCC and ICC. We previously reported that adjuvant HAIC after hepatectomy reduces the risk of recurrence in HCC patients with microvascular invasion (MVI) (21), and preoperative neoadjuvant HAIC can improve the prognosis of patients with potentially resectable BCLC A/B HCC (24). Furthermore, we examined its efficacy and application prospects in unresectable ICC and found that FOLFOX-HAIC exhibited good efficacy and safety for treating unresectable ICC (12). With significant advances in systemic therapy, particularly the extensive use of PD-1/PD-L1 inhibitors in HCC, the continued evaluation of FOLFOX-HAIC along with lenvatinib and durvalumab for the treatment of unresectable ICC is warranted.

The preliminary results of this retrospective study indicated that the triple therapy of lenvatinib and durvalumab combined with FOLFOX-HAIC has a high ORR (39.1%, RECIST; 65.2%, mRECIST) and a considerable median OS (17.9 months) and PFS (11.9 months) in unresectable ICC with acceptable safety profiles. This indicates this novel triple therapy may yield satisfactory efficacy and favorable survival of patients with treatment-naive unresectable ICC.

TKIs synergize with PD-1/PD-L1 inhibitors in liver cancer by targeting VEGFR (25, 26). The high response rate and improved survival observed in our study may be attributed to the synergistic effect of FOLFOX-HAIC, lenvatinib, and durvalumab. HAIC reduces tumor burden by maintaining high concentrations of chemotherapeutic drugs in the tumor. Conversely, immunogenic cell death induced by chemotherapy enhances the antitumor effects of ICIs (27, 28). Lenvatinib can enhance the antitumor activity of durvalumab by inhibiting immunosuppressive cells, such as Tregs, and by promoting the infiltration of immune T cells into the tumor microenvironment (29, 30). Moreover, lenvatinib along with durvalumab may overcome resistance to FOLFOX by disrupting the hypoxic microenvironment within tumors through normalizing the tumor vasculature (31). Therefore, triple therapy with lenvatinib, durvalumab, and HAIC rapidly reduces tumor burden, prolongs systemic treatment response, and further prolongs the long-term survival of patients.

Of note, patients in this study had a median PFS of 11.9 months, which is relatively close to the median OS of 17.9 months. This may be the result of the following: 1) some patients did not return to our center after treatment; thus, we could not assess tumor treatment response. As a result, some patients only had OS data but not PFS data, which introduced some bias in the PFS analysis; 2), ICC often infiltrated along the bile duct because of its high invasiveness, which could rapidly lead to biliary obstruction after progression and cause serious complications, such as obstructive jaundice and pancreatitis. This resulted in a rapid deterioration and death, which may partially explain why the OS is close to the PFS.

In the present study, although nearly all (92.9%) patients experienced varying degrees of AEs, the incidence of severe AEs was similar to that of other studies (8, 18). Leukocytopenia is a common AE associated with chemotherapy (32). In the present study, 32.1% (9/28) of the patients had different degrees of leukocytopenia, of which only 3.6% (1/28) had grade 3–4 AEs. In the TOPAZ-1 trial, the gemcitabine plus cisplatin (GC) regimen along with durvalumab yielded an incidence of grade 3–4 AEs as high as 75.7% (19). However, the incidence of grade 3–4 AEs in our study was only 46.5% (13/28), which was lower compared with that in the TOPAZ-1 trial. This may be because: First, compared with systemic chemotherapy, as a type of local treatment, HAIC directly injected chemotherapeutic drugs into the hepatic artery resulted in a higher local drug concentration and lower extrahepatic non-target drug concentration, effectively reducing AEs, and improving treatment tolerance. Second, our study included only patients with ICC, whereas the TOPAZ-1 study also included patients with ECC and gallbladder cancer, which had a higher incidence of complications and adverse reactions. Overall, the present study used the FOLFOX regimen, whereas the TOPAZ-1 study adopted the GC regimen, so different chemotherapy regimens may also partially explain the differences in AE rates.

There are several limitations to this study. First, this is a retrospective, single-center study; therefore, an unknown selection bias can inevitably be ruled out, which reduces the generalizability of the study. Second, the small sample size and lack of a control group decreased the reliability of the evidence and increased the comparison error. Thus, further studies in a larger population are needed with a control group. Finally, the relatively short follow-up time may reduce the certainty of the observed effectiveness and influence the estimation of ORR, PFS, and OS.

## Conclusion

In summary, FOLFOX-HAIC combined with lenvatinib plus durvalumab showed promising antitumor activity and manageable AEs in patients with treatment-naive unresectable ICC. Although the value of HAIC as a standard of care for unresectable ICC may be limited, this study indicates that this drug combination may be suitable as a novel first-line treatment option for this patient population; however, it should be confirmed in large-scale prospective randomized clinical trials.

## Data availability statement

The original contributions presented in the study are included in the article/**Supplementary Material**. Further inquiries can be directed to the corresponding authors.

## Ethics statement

This study was conducted according to the ethical guidelines of the Declaration of Helsinki and approved by the Ethics Committee (B2020-318-01) of Sun Yat-Sen University Cancer Center (SYSUCC; Guangzhou, China). The studies were conducted in accordance with the local legislation and institutional requirements. Written informed consent for participation was not required from the participants or the participants’ legal guardians/next of kin in accordance with the national legislation and institutional requirements.

## Author contributions

RZ: Writing – original draft. JZ: Writing – original draft. ZM: Data curation, Writing – review & editing. XX: Software, Writing – review & editing. WW: Writing – review & editing. SL: Data curation, Writing – review & editing. RG: Supervision, Writing – review & editing.

## Supplementary material

Supplementary Figure S1 Survival analysis of patients with tumor response and non-response. **(A)** Overall survival and **(B)** Progression-free survival of patients with tumor response and non-response based on the RECIST 1.1 criteria; **(C)** Overall survival and **(D)** Progression-free survival of patients with tumor response and non-response based on the mRECIST criteria.
