# Supplementary figures and images for: Efficacy and safety of lenvatinib plus durvalumab combined with hepatic arterial infusion chemotherapy for unresectable intrahepatic cholangiocarcinoma
Source: Front Immunol. 2024 May 10;15:1397827. doi: 10.3389/fimmu.2024.1397827 (PMC11116590; doi:10.3389/fimmu.2024.1397827)

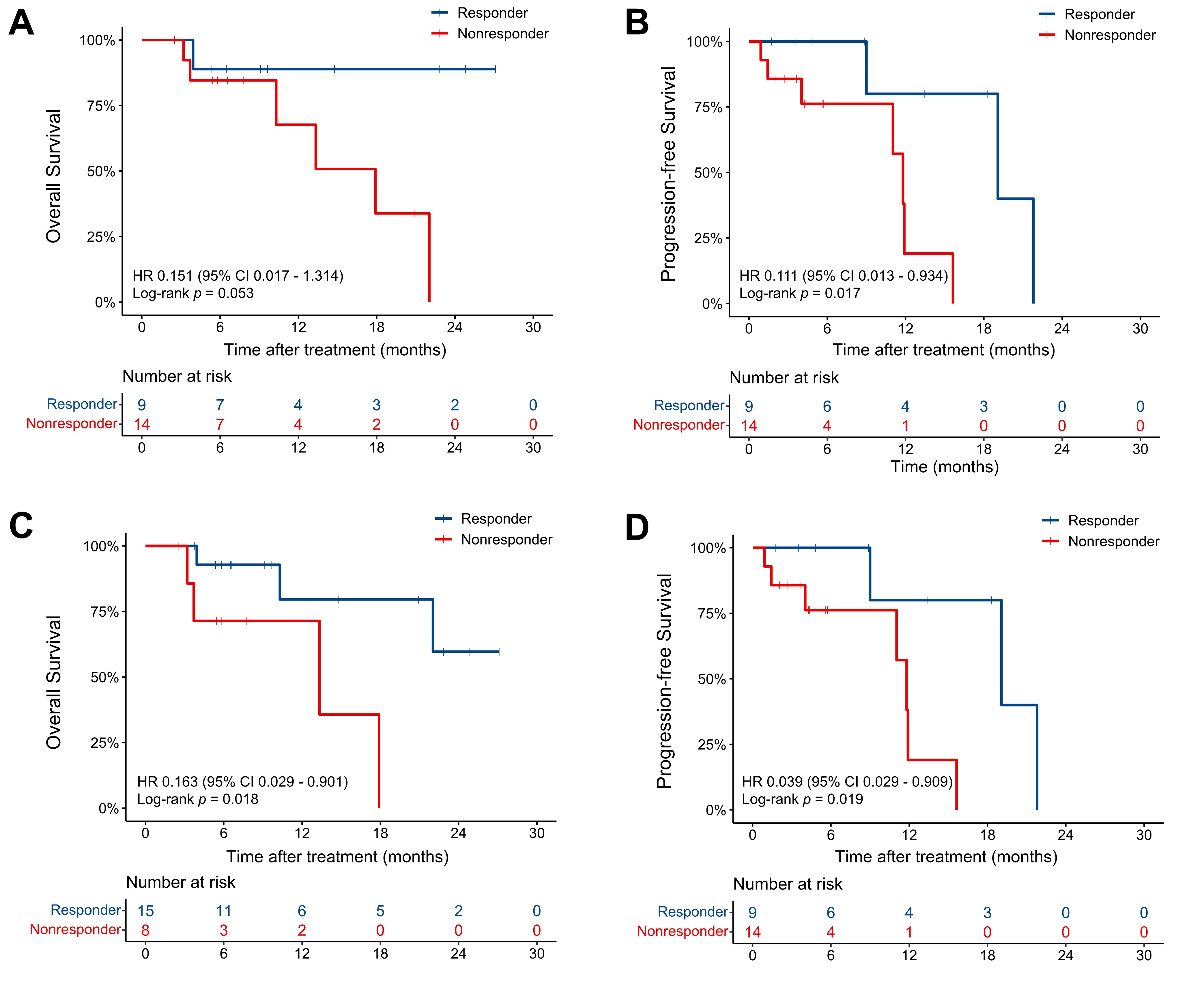

Supplement: Supplementary Figure S1 — Survival analysis of patients with tumor response and non-response. (A) Overall survival and (B) Progression-free survival of patients with tumor response and non-response based on the RECIST 1.1 criteria; (C) Overall survival and (D) Progression-free survival of patients with tumor response and non-response based on the mRECIST criteria. [file Image_1.jpeg]
