# Supplementary material for: Efficacy and safety of lenvatinib plus durvalumab combined with hepatic arterial infusion chemotherapy for unresectable intrahepatic cholangiocarcinoma
Source: Front Immunol. 2024 May 10;15:1397827. doi: 10.3389/fimmu.2024.1397827 (PMC11116590; doi:10.3389/fimmu.2024.1397827)
Supplement: Supplementary file 2 [file DataSheet_1.doc]

**Table S1.** Univariate and multivariate analyses of prognostic factors for overall survival (OS) and progression-free survival (PFS)

| **Characteristics** |  | **Overall survival** | | | | | | **Progression-free survival** | | | | | |
| --- | --- | --- | --- | --- | --- | --- | --- | --- | --- | --- | --- | --- | --- |
|  |  | **Univariate analysis** | | | **Multivariate analysis** | | | **Univariate analysis** | | | **Multivariate analysis** | | |
|  |  | **HR** | **95CI%** | **P** | **HR** | **95CI%** | **P** | **HR** | **95CI%** | **P** | **HR** | **95CI%** | **P** |
| Age (＞50/≤50) | | 1.559 | 0.437 – 5.555 | 0.494 |  |  |  | 2.205 | 0.436 – 9.392 | 0.368 |  |  |  |
| Gender (Female/Male) | | 0.760 | 0.217 – 2.663 | 0.668 |  |  |  | 3.868 | 0.464 – 32.261 | 0.211 |  |  |  |
| ECOG PS (1/0) | | 1.890 | 0.535 – 6.681 | 0.323 |  |  |  | 3.975 | 0.854 – 18.504 | 0.079 |  |  |  |
| CA19-9 (＞40/≤40 U/ml) | | 3.782 | 0.876 – 16.330 | 0.075 |  |  |  | 4.986 | 0.783 – 31.765 | 0.089 |  |  |  |
| HBV (Positive/Negative) | | 0.677 | 0.184 – 2.486 | 0.557 |  |  |  | 0.609 | 0.151 – 2.449 | 0.485 |  |  |  |
| Maximum diameter (＞5/≤5 cm) | | 1.231 | 0.260 – 5.830 | 0.793 |  |  |  | 1.159 | 0.231 – 5.801 | 0.858 |  |  |  |
| TNM stage (III~IV/I~II) | | 1.129 | 0.287 – 4.439 | 0.862 |  |  |  | 0.961 | 0.183 – 5.030 | 0.962 |  |  |  |
| Vascular invasion (Presence/Absence) | | 3.224 | 0.883 – 11.776 | 0.077 |  |  |  | 1.160 | 0.223 – 6.046 | 0.860 |  |  |  |
| LN metastasis (Presence/Absence) | | 1.645 | 0.416 – 6.502 | 0.478 |  |  |  | 0.493 | 0.109 – 2.225 | 0.358 |  |  |  |
| Distant metastasis (Presence/Absence) | | 2.215 | 0.628 – 7.821 | 0.216 |  |  |  | 2.103 | 0.513 – 8.619 | 0.302 |  |  |  |
| Tumor number (Multiple/Single) | | 3.208 | 0.677 – 15.215 | 0.142 |  |  |  | 2.023 | 0.504 – 8.123 | 0.320 |  |  |  |
| Tumor distribution (Bi-lobe/Uni-lobe) | | 1.973 | 0.551 – 7.074 | 0.297 |  |  |  | 1.026 | 0.273 – 3.857 | 0.970 |  |  |  |
| ALT (＞40/≤40 U/ml) | | 2.103 | 0.603 – 7.334 | 0.243 |  |  |  | 0.589 | 0.120 – 2.897 | 0.515 |  |  |  |
| ALB (＞40/≤40 g/L) | | 0.303 | 0.085 – 1.075 | 0.065 |  |  |  | 0.367 | 0.094 – 1.428 | 0.148 |  |  |  |
| TBil (＞17.1/≤17.1 μmol/L) | | 1.890 | 0.484 – 7.385 | 0.360 |  |  |  | 0.654 | 0.079 – 5.379 | 0.693 |  |  |  |
| CRE (＞75/≤75 μmol/L) | | 0.045 | 0.000 – >1000 | 0.740 |  |  |  | 0.041 | 0.000 – >1000 | 0.694 |  |  |  |
| Response (RECIST, responder/non-responder) | | 0.151 | 0.017 – 1.314 | 0.087 |  |  |  | 0.111 | 0.013 – 0.934 | **0.043** | 0.114 | 0.011 – 1.203 | 0.071 |
| Response (mRECIST, responder/non-responder) | | 0.163 | 0.029 – 0.901 | **0.038** | 0.371 | 0.025 – 3.937 | 0.371 | 0.162 | 0.029 – 0.909 | **0.039** | 3.370 | 0.275 – 41.232 | 0.342 |
| Cycles of HAIC (3~4/1~2) | | 0.232 | 0.059 – 0.917 | **0.037** | 0.492 | 0.033 – 5.152 | 0.492 | 0.067 | 0.008 – 0.599 | **0.016** | 0.043 | 0.002 – 0.818 | **0.036** |

ECOG, Eastern Cooperative Oncology Group Performance Status; CA19-9, carbohydrate antigen 19-9; HBV, hepatitis B virus; lymph node; ALT, alanine transaminase; ALB, albumin; TBil, total bilirubin; CRE, creatinine.

RECIST, Response Evaluation Criteria in Solid Tumors; mRECIST, modified RECIST; HAIC, hepatic arterial infusion chemotherapy.

*The font is bolded to emphasize that the *P* value is less than 0.05.
